# Supplementary material for: Fabrication and Optimization of Essential-Oil-Loaded Nanoemulsion Using Box–Behnken Design against Staphylococos aureus and Staphylococos epidermidis Isolated from Oral Cavity
Source: Pharmaceutics. 2022 Aug 5;14(8):1640. doi: 10.3390/pharmaceutics14081640 (PMC9416493; doi:10.3390/pharmaceutics14081640)
Supplement: Supplementary file 1 [file pharmaceutics-14-01640-s001.zip › pharmaceutics-1831498-supplementary.pdf]

Fabrication and optimization of essential oil loaded nanoemulsion using Box-Behnken design against *Staphylococos aureus* and *Staphylococos epidermidis* isolated from oral cavity.

Niamat Ullah[1], Adnan Amin [1*], Rana A. Alamoudi [2], Sheikh Abdur Rasheed* [3], Ruaa A. Alamoudi [4], Asif Nawaz [5], Muhammad Raza[5], Touseef Nawaz [1], Saiqa Ishtiaq [6], Syed Shakil Abbas [7].

Supplementary Information

GCMS Analysis of Clove

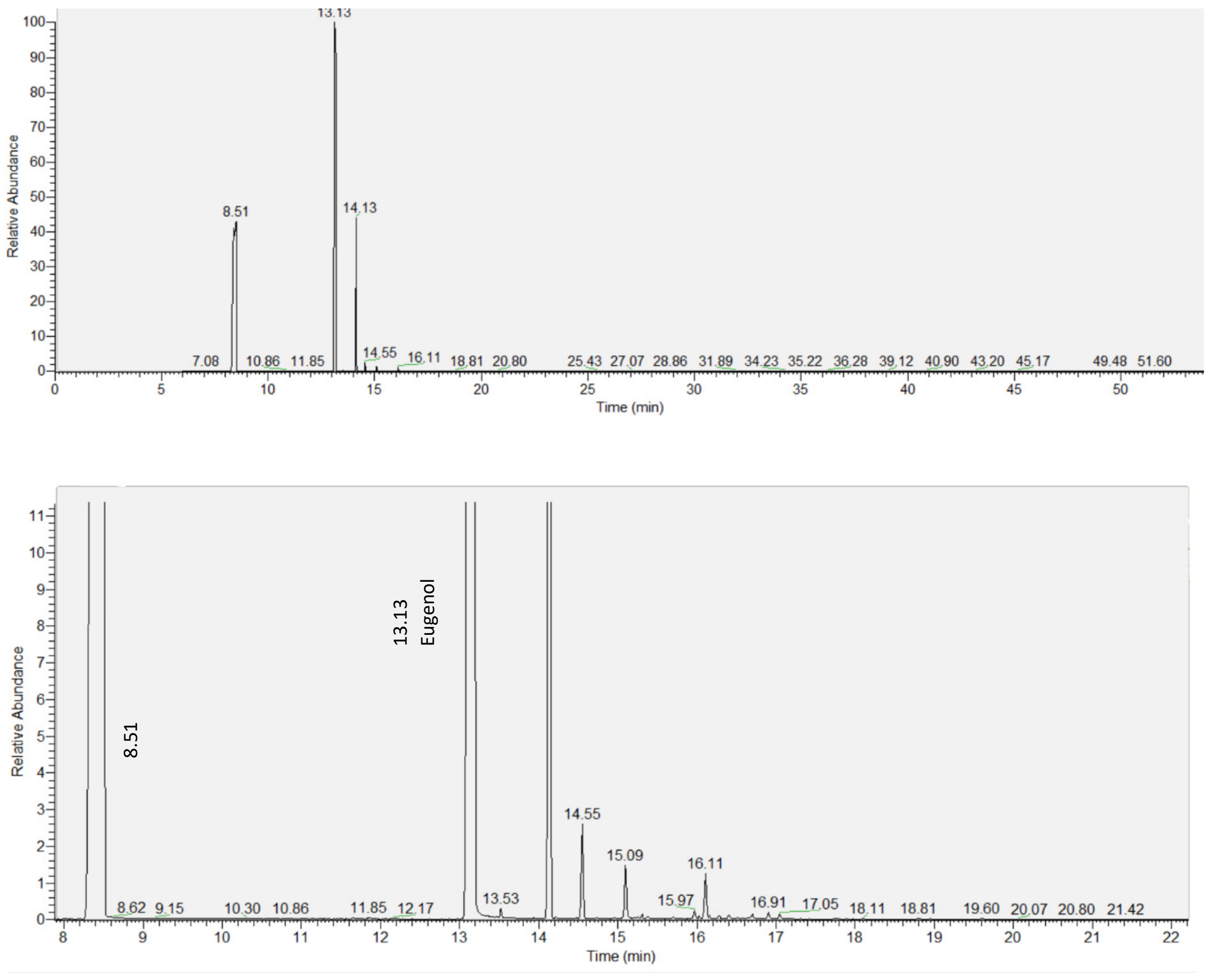

Figure S1. GC-MS Profile of clove essential oil (Adopted from Rafey et al., 2021)

Table: GC-MS analysis of Clove Oil

S.No	Compound	Retention time	RI	Conc %
1	Benzyl alcohol	8.50	1025	traces
2	Methyl salicylate	10.86	1021	0.05
3	Limonene	11.66	1021	traces
4	Cubebene	11.86	1022	traces
5	**Eugenol**	**13.13**	**1132**	**75**
6	Iso-eugenol	13.53	1011	11.08
7	Caryophyllene	14.13	1322	10.2
8	α-Humulene	14.55	1422	1.22
9	Eugenol acetate	15.09	1357	12.2
10	2-carene	16.11	traces	traces
11	β-Humulene	16.28	1021	0.84
12	Cubenol	16.40	1024	0.02
13	α-Farnesene	16.70	1254	0.21
14	Caryophylene oxide	16.91	1233	0.34

(Adopted from Rafey et al., 2021)

Table GCMS component Analysis of Cinnamon oil

S.No	Compound	Retention time	RI	Conc %
1	α-Pinene	5.66	899	1.3
2	Benzaldehyde	6.40	963	0.3
3	p-Cymene	7.82	1025	1.9
4	Limonene	7.93	1075	1.2
5	Eucalyptol	**8.08**	**1084**	**5.4**
6	c-Terpinene	8.66	1121	0.4
7	Linalool	9.86	1188	7
8	Isoborneol	11.64	1275	0.8
9	**(E)-cinnamaldehyde**	**15.22**	**1414**	**71.5**
10	Eugenol	16.90	1469	4.6
11	β-Caryophyllene	18.58	1518	6.4
12	Acetic acid, cinnamyl ester	19.23	1536	0.5
13	α-Humulene	19.47	1543	1.7
14	δ-Cadinene	20.97	1581	1.4
15	trans-Calamenene	21.10	1585	0.7
16	Caryophyllene oxide	22.61	1621	0.5
17	Benzyl benzoate	26.82	1710	0.5

(Adopted from Alizadeh Behbahani et al., 2020)

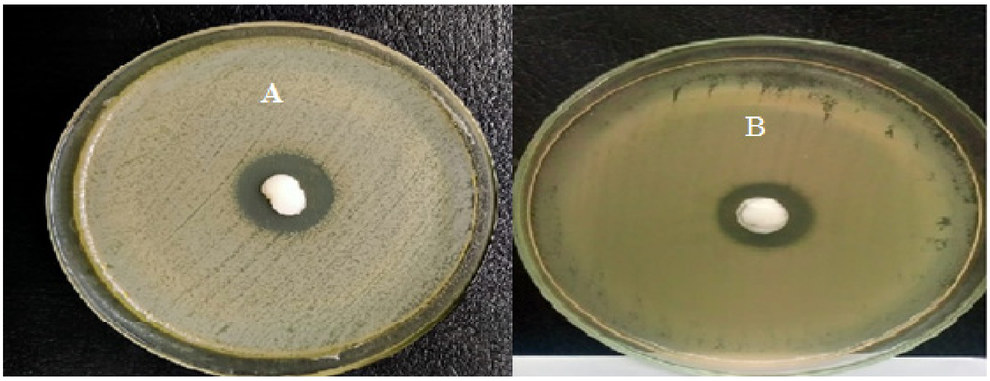

Figure S2: (A) antibacterial activity of essential oil loaded nanoemulsion against *S.aureus*(B) antibacterial activity against *S.epidermidis*.

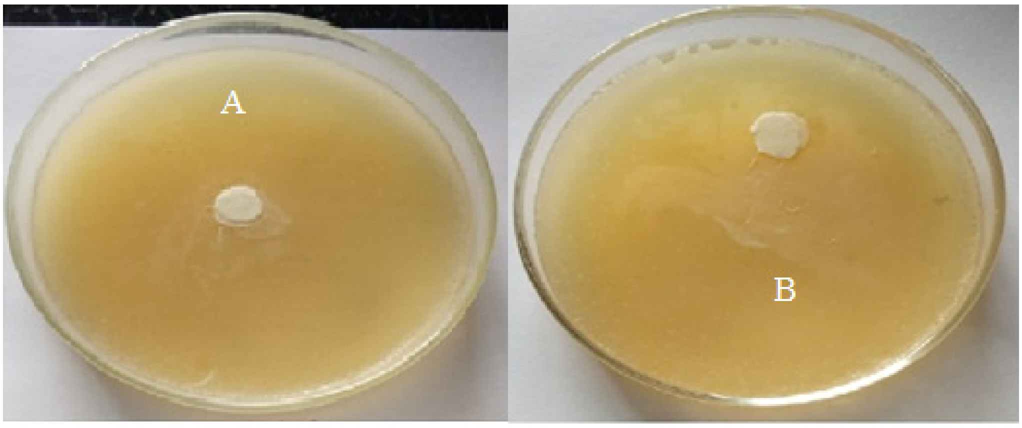

Figure S3: (A) antibacterial activity of unloaded nanoemulsion against *S.aureus* (B) antibacterial activity of unloaded nanoemulsion against *S.epidermidis.*

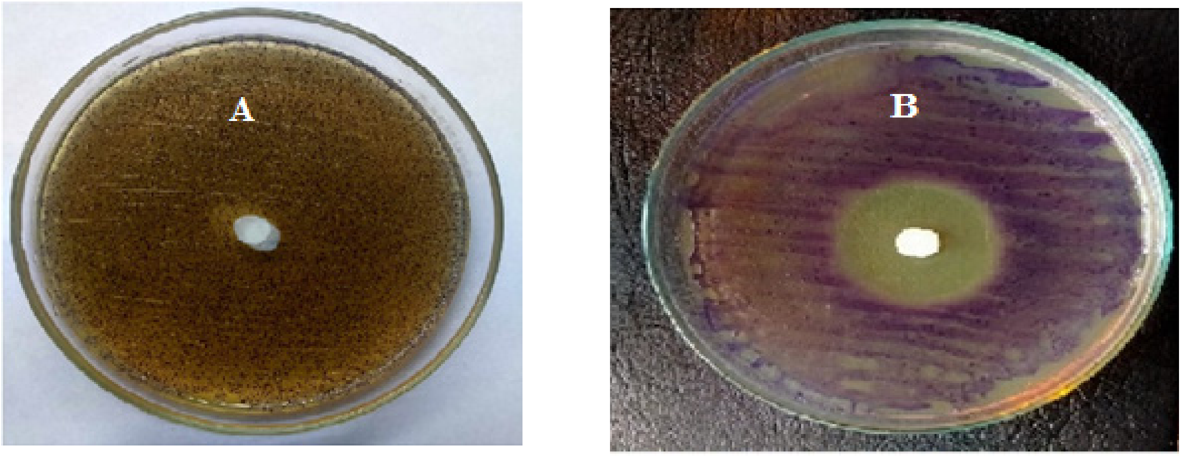

Figure S4: (A) Antiquorum sensing activity of unloaded nanoemulsion and (B) Antiquorum sensing activity of essential oil loaded nanoemulsion.

References

Rafey A., Amin A., Kamran M., Haroon U., Farooq K., Foubert K., Pieters L. Plant Origin Antibiotics Against Periodontal Infections; Antibiofilm, Anti-Quorum Sensing, Molecular Docking Studies and Characterization Of Active Constituents". *Antibiotics* 2021, *10*(12), 1504.

Alizadeh Behbahani B, Falah F, Lavi Arab F, Vasiee M, Tabatabaee Yazdi F. Chemical Composition and Antioxidant, Antimicrobial, and Antiproliferative Activities of *Cinnamomum zeylanicum* Bark Essential Oil. *Evid Based Complement Alternat Med*. 2020;2020:5190603. Published 2020 Apr 29. doi:10.1155/2020/5190603
